# XTEN as Biological Alternative to PEGylation Allows Complete Expression of a Protease-Activatable Killin-Based Cytostatic

**DOI:** 10.1371/journal.pone.0157193

**Published:** 2016-06-13

**Authors:** Akvile Haeckel, Franziska Appler, Angela Ariza de Schellenberger, Eyk Schellenberger

**Affiliations:** 1 Department of Radiology, Charité - Universitätsmedizin Berlin, Berlin, Germany; 2 nanoPET Pharma, Berlin, Germany; Tecnologico de Monterrey, MEXICO

## Abstract

Increased effectiveness and reduced side effects are general goals in drug research, especially important in cancer therapy. The aim of this study was to design a long-circulating, activatable cytostatic drug that is completely producible in *E*. *coli*. Crucial for this goal was the novel unstructured polypeptide XTEN, which acts like polyethylene glycol (PEG) but has many important advantages. Most importantly, it can be produced in *E*. *coli*, is less immunogenic, and is biodegradable. We tested constructs containing a fragment of Killin as cytostatic/cytotoxic element, a cell-penetrating peptide, an MMP-2 cleavage site for specific activation, and XTEN for long blood circulation and deactivation of Killin. One of three sequence variants was efficiently expressed in *E*. *coli*. As typical for XTEN, it allowed efficient purification of the *E*. *coli* lysate by a heat step (10 min 75°C) and subsequent anion exchange chromatography using XTEN as purification tag. After 24 h XTEN-Killin reduced the number of viable cells of HT-1080 tumor cell line to 3.8 ±2.0% (p<0.001) compared to untreated controls. In contrast, liver derived non-tumor cells (BRL3A) did not show significant changes in viability. Our results demonstrate the feasibility of completely producing a complex protease-activatable, potentially long-circulating cytostatic/cytotoxic prodrug in *E*. *coli*—a concept that could lead to efficient production of highly multifunctional drugs in the future.

## Introduction

Developing new cytostatic drugs with enhanced anticancer effectiveness but reduced side effects in non-cancerous tissues is still the central goal of cancer research. One strategy to achieve this goal is to modulate the biodistribution of active drugs by reducing their accumulation in critical organ tissues sensitive to side effects while enhancing their uptake into malignant tumors. This can be accomplished by enhancing the hydrophilic properties through coupling of polymers like polyethylene glycol (PEG) to the active agent in order to reduce the unspecific uptake by normal tissues, which additionally can increase the circulation time in blood and the interval between drug applications. Furthermore, uptake of PEGylated substances into tumors is promoted by the enhanced permeability and retention (EPR) of tumors resulting from leaky tumor blood vessels and altered lymphatic vessels. This so-called EPR effect facilitates the transport and retention of macromolecules (larger than 40 kDa) in tumor tissues [1,2] and has been reported to reduce side effects [3–5]. A clinical example is a PEGylated liposome-encapsulated form of doxorubicin (Doxil), which has lower cardiac toxicity [6]. However, even though immunogenicity of PEGylated drugs is considered to be very low, repeated treatment can result in induction of antibodies against PEG [7]. Moreover, antibodies against PEG were found in 22–25% of healthy blood donors up from 0.2% two decades ago, which could be the consequence of increasing use of PEG in cosmetics, therapeutics, and processed food [8].

Another way to reduce side effects is to develop inactive prodrugs that are specifically activated in the tumor area. The inclusion of cleavage sites of proteases that are highly expressed in tumors, e.g. matrix metalloproteinases MMP-2 and MMP-9, provides an option for building activatable drugs [9]. For example, doxorubicin was coupled to a MMP 2/9-cleavable peptide resulting in a therapeutically inactive prodrug. When injected, this prodrug could be activated by the increased MMP activity in the tumor, whereas the toxicity to other tissues, particularly the heart, was reduced [10].

To achieve substantial tumor uptake of drugs that do not penetrate plasma membranes themselves, so-called cell-penetrating peptides (CPP) [11], also known as protein transduction domains (PTDs), have been developed. Such peptides, derived from proteins (penetratin, Tat peptide), as well as synthetic polyarginines have been shown to efficiently deliver biologically active molecules into the cell [12].

The aim of this study was to develop a fusion protein that combines the aforementioned features in a way that allows complete synthesis in *E*. *coli*.

The key to accomplishing this goal was to replace PEG by the unstructured hydrophilic polypeptide XTEN, which combines the desirable properties of PEG such as longer blood half-life and stabilization of proteins with several additional decisive advantages: it is biodegradable, less immunogenic than PEG, it has defined masses and does not need to be chemically coupled, but instead, can be expressed together with the effector protein and used as purification tag [13]. Besides prolonging the blood half-life of exenatide with XTEN, other examples are XTEN versions of glucagon [14], of glucagon-like peptide 2 (GLP2) [15], and XTEN-annexin A5 developed by us for imaging apoptosis [16]. For finding an expressible cytostatic fusion protein and keeping the costs for DNA syntheses low we chose an XTEN variant of 288 amino acids. When a suitable fusion protein variant is found the short XTEN can be exchanged with a longer XTEN for *in vivo* application, e.g. 864 amino acids for longer blood circulation times [13].

As the cytostatic (cell division stopping) and cytotoxic (apoptosis inducing) component, we chose a partial sequence of Killin (KLLN), which was recently discovered and is regulated by PTEN (phosphate and tensin homologue). PTEN is a very well characterized tumor suppressor gene that is regulated by p53 and reduces the phosphoinositol-3-kinase/protein kinase B (Akt) level, thus inducing G1 cell cycle arrest and apoptosis [17]. Mutations of this gene are associated in most cases with Cowden syndrome (CS) and hence with a high risk of developing breast, thyroid, and endometrial cancer [18]. Killin, which shares the transcription start site with PTEN, is regulated by the same promoter, but, surprisingly, is transcribed in opposite direction [19]. It has been reported that Killin, as DNA-binding protein and tumor suppressor, is involved in S-phase cell cycle arrest and induction of apoptosis of many cancer cell types and may be regulated by p53 [19, 20]. Interestingly, according to Bennett *et al*. 2011, about 30% of CS patients without PTEN mutations had hypermethylation and downregulation of the Killin gene [10]. In prostate cancer, Killin was shown to decrease prostate-specific antigen levels and suppress androgen-mediated cell growth by inhibiting androgen receptor (AR) transcription [21]. Killin was suggested as an anticancer drug but expression as recombinant protein in *E*. *coli* and purification were considered to be virtually impossible [19].

Thus the recombinant therapeutic fusion protein included *(1)* Killin as cytostatic/cytotoxic component, *(2)* XTEN for long blood circulation time, deactivation of Killin and passive targeting of the tumor by exploiting the EPR effect, *(3)* an MMP2/9 cleavage site for specific activation in the tumor, and *(4)* a CPP to transfer Killin into the cells. Deactivation was fundamentally important not only for function, but also for the expression in *E*. *coli*, since active Killin is too toxic for *E*. *coli* cells [19] to be produced in significant amounts.

## Materials and Methods

### Chemicals

Kanamycin was purchased from Carl Roth (Karlsruhe, Germany). LB agar, LB medium, MagicMedia *E*. *coli* expression medium, Novex 4–12% Bis-Tris gradient gels, Coomassie SimplyBlue SafeStain, fetal bovine serum, phosphate buffered saline (PBS), penicillin and streptomycin were obtained from Life Technologies (Darmstadt, Germany). BugBuster protein extraction reagent, Benzonase endonuclease and MMP-2 enzyme were purchased from Merck Millipore (Darmstadt, Germany), and Proteinase Halt protease inhibitor cocktail, maleimide-fluorescein, and bicinchoninic acid (BCA) protein assay were obtained from Thermo Fisher Scientific (Schwerte, Germany). BioGel P6 was bought from Bio-Rad Laboratories GmbH (Munich, Germany). Diethylaminoethyl (DEAE) cellulose, Octyl-Sepharose 4 Fast Flow and camptothecin were bought from Sigma-Aldrich (Steinheim, Germany). Maleimide-6S-IDCC was purchased from Mivenion GmbH (Berlin, Germany). Dithiothreitol (DTT), 4-(2-hydroxyethyl)-1-piperazineethanesulfonic acid (HEPES), ethylene diamine tetraacetic acid (EDTA), trifluoroacetic acid (TFA), sodium carbonate, sodium thiosulfate, silver nitrate and all other chemicals were purchased from Sigma-Aldrich (Steinheim, Germany).

### Expression and purification of XTEN-Killin

The coding sequence was built by fusing the following DNA components: an XTEN variant of 288 amino acids (XTEN288) [13], 8 x glutamate as counterpart to arginines of the CPP, MMP2/9 cleavage sequence (low cleavage by neprilysin [22]), a Killin fragment with cytostatic activity (deletion mutant 8–49 aa, 4,9 kDa [19]), 6 x arginine (CPP) followed by one cysteine residue, which allows subsequent specific labeling because it is the only cysteine in the sequence. Gene synthesis, subcloning and transformation of *E*. *coli* cells with subsequent culturing in auto-induction medium were performed as described before [16].

Bacterial cells were then collected by centrifugation and lysed in BugBuster protein extraction reagent according to the manufacturer’s instruction. The bacterial cell lysate was heated to 75°C for 10 min and cleared by centrifugation at 20,000 g for 30 min, and applied to a 50 ml weak anion exchange column DEAE (diethylaminoethyl) cellulose, equilibrated with the starting buffer (20 mM Tris, 50 mM NaCl, pH = 6.8). The protein of interest was eluted using a gradient to end buffer (20 mM Tris, 500 mM NaCl, pH = 6.8) with a flow rate of 2 ml/min using a BioLogic LP system (BioRad, Munich, Germany). The fusion protein-containing fractions were identified by SDS-PAGE (Novex 4–12% Bis-Tris gradient gel) with subsequent Coomassie safe stain and pooled. After buffer exchange to 20 mM Tris, 1.7 M ammonium sulfate, pH = 7.2, this solution was loaded on a 30 ml Octyl-Sepharose 4 Fast Flow hydrophobic interaction column, and the desired protein was eluted using a gradient to end buffer (20 mM Tris, pH = 7.5). XTEN-Killin fractions were selected again as described above, then pooled and desalted against storing buffer (10 mM HEPES, 135 mM NaCl, pH 7.5). The protein solution was passed through a 0.22 μm filter device. The correction factor for the BCA assay was calculated as described [16].

### HPLC and coupling of XTEN-Killin with NIRF dye 6S-IDCC and fluorescein

HPLC analysis of the purified XTEN-Killin and labeling of the recombinant protein with near-infrared fluorescence (NIRF) dye maleimide-6S-indodicarbocyanine (IDCC) and maleimide-fluorescein were performed using a reversed phase column (Acclaim 300 RP-C18, Dionex, USA) as described before [16].

### Enzymatic digestion of XTEN-Killin by MMP-2

The solution containing 8 μg of XTEN-Killin, treated before with 10 mM DTT to break dimers, was diluted 1:1 with digestion buffer, and 0.1 ng/μl of MMP-2 was added or left out for control. After 2 h incubation at 37°C, both samples were analyzed using SDS-PAGE, Coomassie stain, and silver stain. For silver stain, the gel was rinsed with bidest water and fixed in 50% methanol and 7% acetic acid for 45 min at room temperature. After this the gel was washed overnight in bidest water. Further followed sensitization step (0.02% sodium thiosulfate for 2 min). The gel was washed 2 times for 1 min in bidest water, incubated with chilled 0.1% silver nitrate at 4°C for 30 min in the dark, washed again 2 times for 1 min in bidest water and developed in 2% sodium carbonate solution containing formaldehyde (0.6 μl/ml of 37% solution).

### Flow cytometry and fluorescence microscopy imaging

#### Cell lines

Human T-cell leukemia (Jurkat) and HeLa cells were purchased from the Leibniz Institute DSMZ-German Collection of Micro-organisms and Cell Cultures, BRL-3A—from I.A.Z. (Munich) and HT-1080 cells were bought from ATCC. Jurkat cells were grown in RPMI 1640 medium containing GlutaMAX (Gibco), HT-1080 and HeLa cells—in EMEM (ATCC), BRL-3A cells—in DMEM/F12 (Gibco). All media were supplemented with 10% fetal bovine serum, 100 U/ml penicillin and 100 mg/ml streptomycin (Life Technologies, Darmstadt, Germany) at 37°C with 5% CO_2_. Before treatment, cells were seeded at a density of 15,000/cm^2^ and left to adhere for 6–8 h.

#### Flow cytometry and statistics

For positive-control of apoptosis, cells were treated with camptothecin for 4 h at 37°C with 5% CO_2_. For analysis of apoptosis, the cells were harvested, centrifuged at 1000 g for 5 min, and washed in cold phosphate-buffered saline (PBS). After a repeated centrifugation step, the cells were resuspended in cold binding buffer (10 mM HEPES, 150 mM NaCl, 5 mM KCl, 1 mM MgCl_2_, and 1.8 mM CaCl_2_, pH 7.4) and incubated with 0.2 μg Annexin A5-fluorescein or Annexin A5-6S-IDCC for 10 min in the dark at room temperature. Subsequently, cells were centrifuged again, and measurements were performed using a C6 Accuri flow cytometer (BD Biosciences, Heidelberg, Germany) at excitation lengths of 488 nm (FITC) and 633 nm (NIFR dye). The results were analyzed using the BD Biosciences C6 Accuri software.

All measurements were performed using the same buffer volume for all cell groups. At least 10,000 cells were counted, cell debris and other particles were excluded according to FSC/SSC measurements. To determinate relative cell density, mean density of treated cells was compared with mean density of control cells, which was equaled to 100%, to obtain percentage value ± standard error of the mean (SEM). Three independent experiments were done for HT-1080 and HeLa, and two for BRL-3A cell groups. Apoptotic cells were identified as annexin A5 positive. Graphic and statistic analysis (one-way ANOVA with Tukey’s multiple comparison test) was done using Prism software (GraphPad Software, Inc., USA).

#### Standard microscopic and confocal fluorescence imaging

For microscopic imaging, adherent HT-80 cells were washed with PBS, fixed in 2% paraformaldehyde for 5 min at room temperature, or detached with trypsin, stained using an annexin A5 assay and imaged without fixation. Jurkat cells growing in suspension were imaged directly, or previously stained with annexin A5 (as mentioned above). Analysis was performed using an Axio Observer.Z1 fluorescence microscope equipped with an ApoTome device (Carl Zeiss AG, Jena, Germany) for confocal imaging.

## Results

### Expression in *E*. *coli*, purification, and characterization of recombinant XTEN-Killin

**Fig 1:**
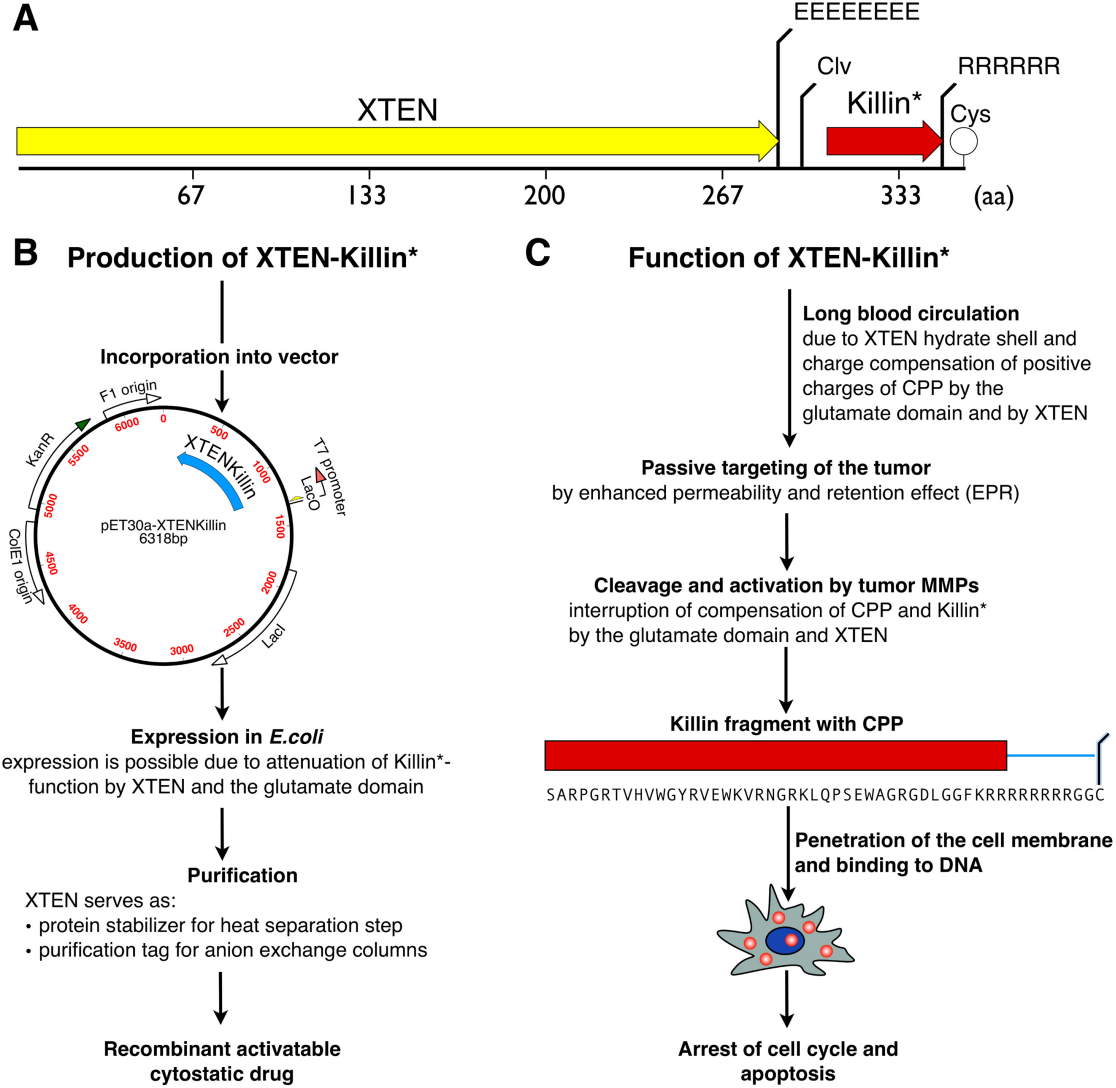
Construction of the recombinant cytostatic/cytotoxic protein XTEN-Killin. Schematic representation of the DNA/protein sequence of XTEN-Killin (A), the procedure for production (B), and a summary of its intended functions (C).

For the design of the successfully expressible construct, an XTEN sequence of 288 amino acids was fused through a glutamate chain of 8 negatively charged amino acids (the neutralizing part of CPP), followed by an MMP2/9 cleavage site, to a active domain of Killin (Killin*–amino acids 8 to 49) as described by Cho *et al*. *2008* [19] (Fig 1 and Figure D in S1 File). The structures of two other variants of the fusion protein, where XTEN was located at the C-terminus, could not be expressed in *E*. *coli* and are given in the Supporting Information section (Figure C in S1 File). Two arginine amino acids at the C-terminal end of Killin, together with an additional 6 arginines, constitute the positive part of a CPP, whose charges are countered by the glutamate chain. This polyarginine CPP was chosen because it has been reported to make the escape from endolysosomal structures 1.7 times more efficient compared with other HIV-derived peptides, and has been discovered to cross cell membrane, e.g., PTD-TAT [23].

The specific cleavage site (PLGLYL) we chose for MMP-2 and MMP-9 was a sequence described by Albright *et al*. 2005 [22], which has low cleavage/activation by non-tumor proteases, especially by neprilysin, a membrane metalloendopeptidase highly expressed throughout the body. A single cysteine at the C-terminus of the fusion protein serves as coupling site for thiol-based labeling. Overall the size of the fusion protein was 358 amino acids with a calculated weight of 34.6 kDa and a calculated isoelectric point of 3.74.

Plasmids encoding the recombinant XTEN-Killin were expressed in *E*. *coli* as described before [16] (see also Materials and Methods). Next, almost 80% of endogenous proteins of *E*. *coli* could be removed by heating the whole-cell lysate for 10 min at 75°C. This heating step is possible, because the XTEN sequence can substantially increase the thermal stability of the fused proteins [13].

**Fig 2:**
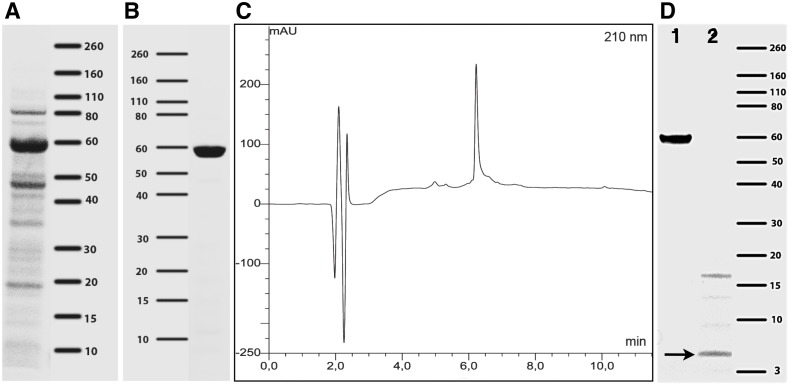
Expression, purification, and cleavage of XTEN-Killin. (A-C) Bacterial lysate expressing XTEN-Killin after heating step (A) and purified XTEN-Killin (B) were analyzed with SDS-PAGE, followed by Coomassie staining. The protein solution was additionally analyzed with HPLC (C) at 210 nm, revealing purity of >90% (peak at 6.3 min). (D) XTEN-Killin coupled with near-infrared dye 6S-IDCC was also analyzed with SDS-PAGE followed by Coomassie staining before (1) and after (2) digestion with MMP-2 enzyme to prove the functionality of the cleavage site. Free XTEN can not be stained with Coomassie, therefore after digestion (2) only CPP killin (approx. 5 kDa, black arrow) and potential complexes formed by the arginine-rich composition of the CPP Killin are visible (approx. 17 kDa). Some additional thin bands at 9 and 13 kDa could represent degradation products after digestion. Markers in A, B and D: 3–260 kDa.

The cleared lysate (Fig 2A) was further purified to remove remaining host cell contaminations and possible degradation products by anion exchange chromatography followed by an additional hydrophobic interaction chromatography (Fig 2B, see also supplementary Figure A in S1 File) for chromatograms and Materials and Methods for detailed protocols). The XTEN-Killin product exhibited relatively slow migration in SDS-PAGE with an apparent molecular weight of about 60 kDa instead of the expected 35 kDa. This is typical for XTEN fusion proteins and can be explained by a large hydration shell, which lets XTEN fusion proteins appear as larger proteins in protein gels [13,16] (Fig 2A–2C). Expression yield of XTEN-Killin in the heated lysate was about 18 mg / 1 L medium. First purification column resulted in lost of 30%, the second in a lost of approximately 25% as calculated by analysis of SDS-PAGE (area and intensity of bands). The final protein yield was 8 mg / 1L medium as determined by BCA. Purification was further analyzed by HPLC, which revealed a purity of 92% and about 8% remaining impurities, e.g. degradation products (Fig 2C). The calculation of a BCA correction factor, which is necessary due to the unusual amino acid content of XTEN, was done as described before [16] and yielded a value of 1.8.

To demonstrate the functionality of the cleavage site and to confirm the sequence, XTEN-Killin-6S-IDCC was digested with MMP-2 (Fig 2D). Complete digestion with MMP-2 occurred within 2 h at 37°C, confirming the correct cleavage site and accessibility for enzyme: in lane 2, only digestion products without XTEN fragment are visible: CPP killin (approx. 5 kDa) and potential aggregates formed by the arginine-rich composition of the CPP Killin. Due to the lack of aromatic amino acids, free XTEN polypeptide cannot be stained by Coomassie stain, but can be visualized by silver stain (Figure B in S1 File). However, complete digestion with MMP-9 enzyme was seen only after 24 h at 37°C (data not shown).

### MMP-dependent cellular uptake of fluorescent XTEN-Killin

**Fig 3:**
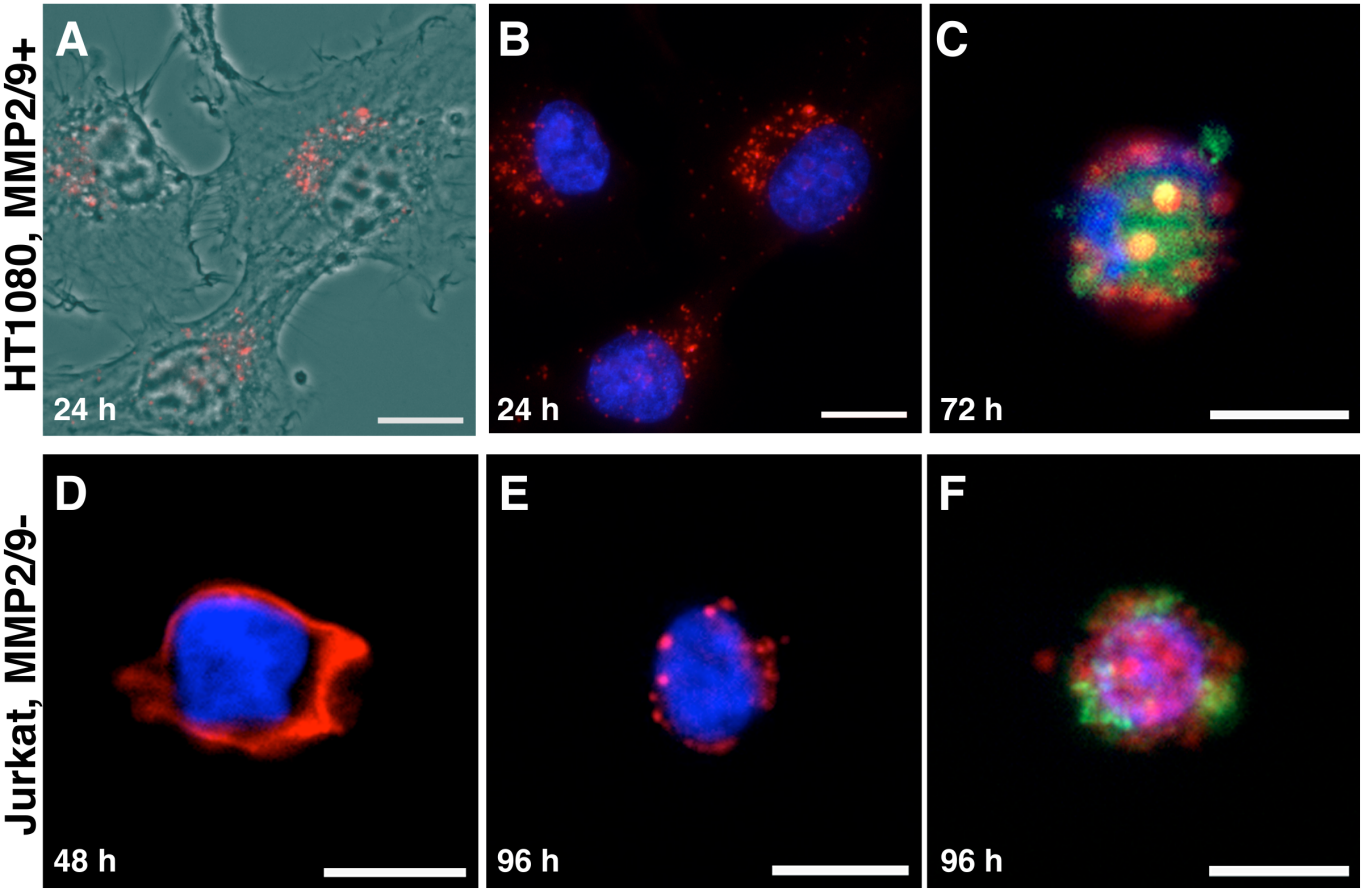
Uptake of XTEN-Killin-6S-IDCC in cancer cells. Low density epithelial sarcoma (HT-1080) cells were exposed to 3 μM (A and B) and 5 μM (C) XTEN-Killin-6S-IDCC for 24–96 h. After 24 h, intracellular enrichment of likely cleaved Killin-6S-IDCC in the endosomes of the MMP-2-positive HT-1080 cells was visible (red, A-B). T-cell lymphoma (Jurkat) cells, which are characterized by low MMP-2 expression, were exposed to 5 μM XTEN-Killin-6S-IDCC for 24–96 h. Up to 48 h, Jurkat cells showed only extracellular binding of the XTEN-Killin-6S-IDCC to the membrane (D, red). Intracellular uptake was observed only at 96 h (E, red). 72 h after treatment of HT-1080 cells (C) and 96 h after treatment of Jurkat cells (F), only a few Killin-6S-IDCC-positive cells (red, intranuclear) were found positive for annexin A5, used as assay for apoptosis/cell death (green). Blue color—DAPI stain. Bars: 10 μM.

**Fig 4:**
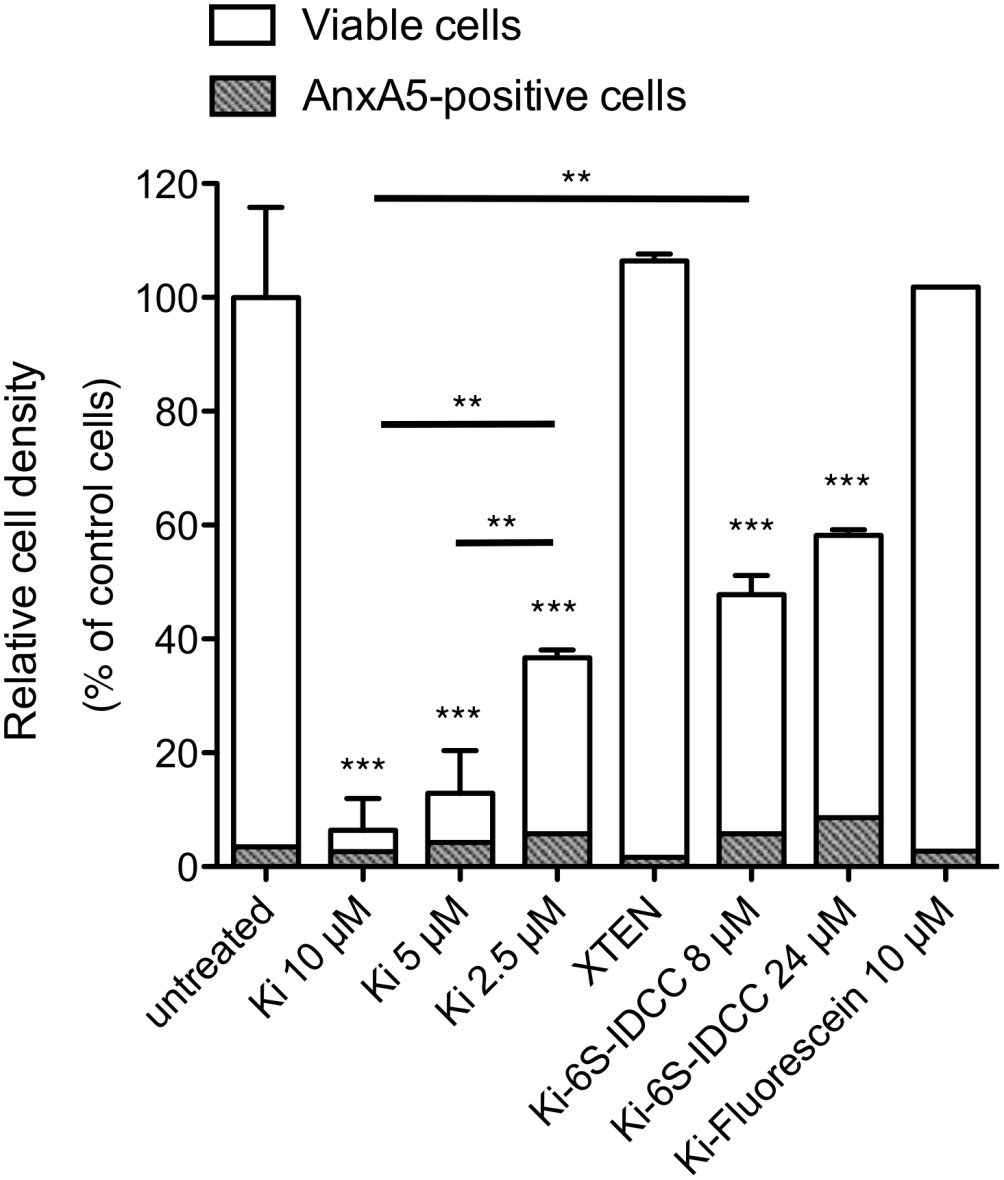
Dose-dependent toxicity of XTEN-Killin. HT-1080 cells were treated with different amounts of XTEN-Killin (Ki) or XTEN-Killin-6S-IDCC (Ki-6S-IDCC) and XTEN-Killin-fluorescein (Ki-fluorescein), as well as XTEN polypeptide, and analyzed by flow cytometry. Relative cell densities of viable and apoptotic annexin A5 positive cells (± SEM) were compared to untreated control cells (100% ±SEM) after 24 h of treatment. At least 3 independent experiments for each group were analyzed using one-way ANOVA with Tukey’s multiple comparison test; *** p<0.001, ** p<0.01. The experiment with XTEN-Killin-fluorescein (Ki-fluorescein) was done without repetitions.

For visualization by fluorescence, the purified XTEN-Killin fusion protein was labeled with maleimide-6S-IDCC dye at the singular cysteine and yielded a labeling efficiency of 89%. Coupling of XTEN-Killin with maleimide-fluorescein was done accordingly. To observe the delivery of XTEN-Killin into cells, human fibrosarcoma HT-1080 cells and human lymphoma Jurkat T-cells were incubated in culture medium with 3 μM XTEN-Killin-6S-IDCC. The HT-1080 cell line has been shown to express high basal levels of MMP-2 and MMP-9, while Jurkat cells, in contrast, exhibit low basal levels of MMP-2 and MMP-9 as reported by Roomi *et al*. [24] and confirmed by our observations (data not shown). Thus the cleavage of XTEN-Killin-6S-IDCC and uptake of the resulting Killin-6S-IDCC domain should be more effective in HT-1080 cells compared to Jurkat cells. As expected, the MMP-2-positive HT-1080 cells demonstrated substantial uptake 24 h after treatment mainly in endosomal structures, but also in the nucleus (Fig 3A and 3B). In contrast, even after 48 h, only membrane-bound XTEN-Killin-6S-IDCC was observed for the MMP-2-negative Jurkat cells, which appeared in endosomal intracellular structures after 96 h, suggesting that the lower MMP-2 expression resulted in slower cleavage and activation of the fusion protein (Fig 3D–3F). After 72 h for HT1080 and after 96 h for Jurkat cells, we detected only a few Annexin A5- positive and therefore apoptotic or necrotic cells. Treatment of both cell types with 3–5 μM concentrations of XTEN-Killin-6S-IDCC had no considerable effect on cell growth. Even higher concentrations of 8–24 μM protein caused only reduced cell growth without signs of massive death (Fig 4).

A few apoptotic cells, confirmed with the annexin binding assay, were observed on confocal fluorescent images (Fig 3C and 3F), exhibiting accumulation of fluorescent killin in the nucleus. To test whether endosomal escape of cell-penetrating peptides and their cargos was the limiting factor [25] and to improve delivery of killin fragment to the nucleus, coincubation of the cells with XTEN-Killin-6S-IDCC and TAT-HA2 was performed as described [26,27]. However, this procedure did not improve the cytostatic effect of labeled XTEN-Killin-6S-IDCC (data not shown). Flow cytometry analysis of HT-1080 and Jurkat cells confirmed the results of fluorescence microscopy imaging (staining with annexin A5, data not shown).

### Unlabeled XTEN-Killin causes strong growth arrest and apoptosis of cancer cells

Unlike XTEN-Killin-6S-IDCC, XTEN-Killin without coupled fluorescence dye dramatically decreased growth of the cancer cell line HT-1080 in a concentration-dependent manner and induced strong apoptosis as determined by flow cytometry (Fig 4): 10 and 5 μM concentration of XTEN-Killin caused stable growth arrest of seeded cells (about 5% of control within 24 h) and rising apoptosis, which peaked (> 40%) within 48 h. In comparison, fluorescent XTEN-Killin-6S-IDCC only decreased cell growth by about 50% even at higher concentrations of 8 μM and 24 μM. Killin-FITC caused no changes in cell growth or viability. XTEN polypeptide alone had no effect on the cell growth rate, and the level of apoptosis was similar to that of untreated control cells.

**Fig 5:**
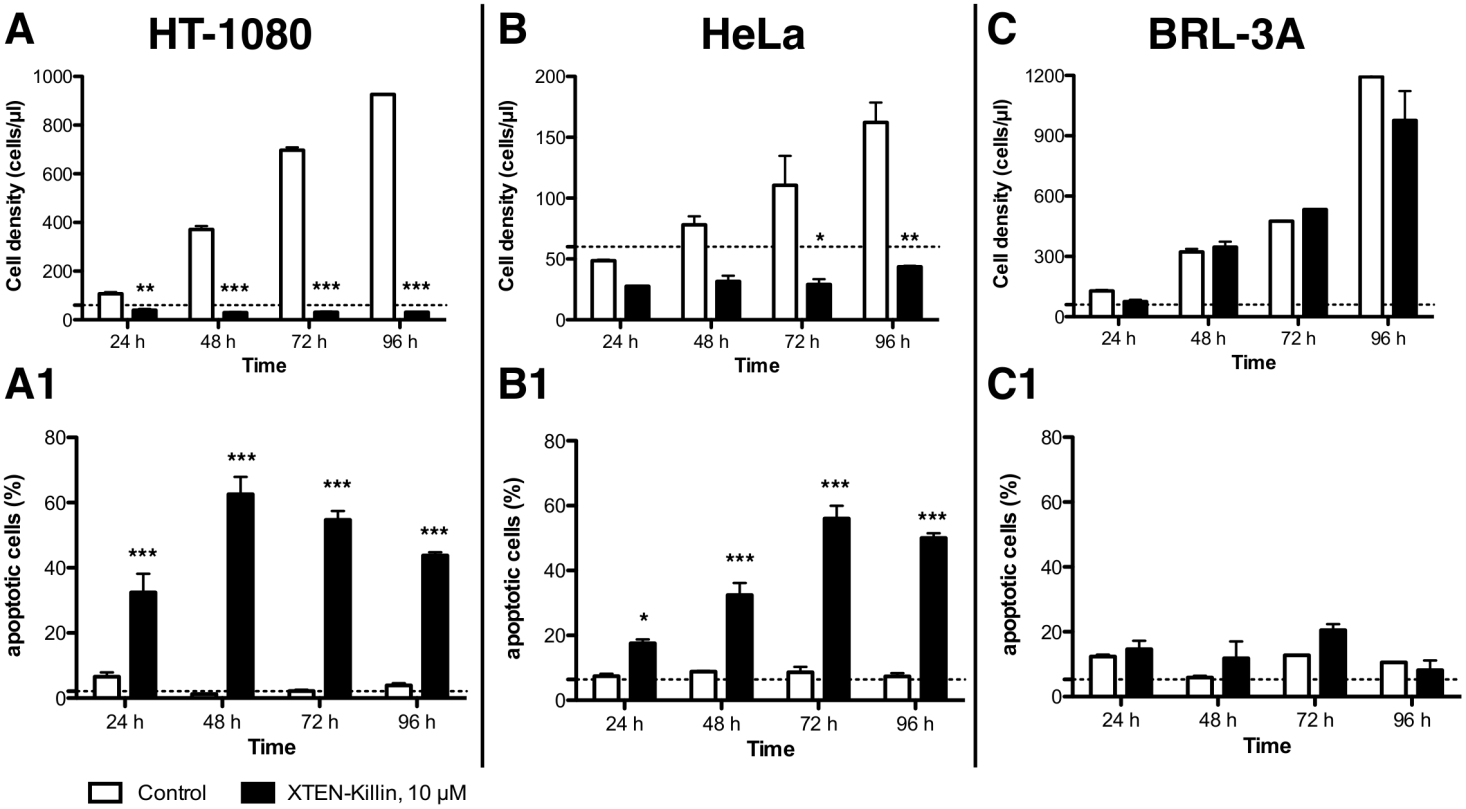
Treatment effects of XTEN-Killin on normal and cancer cells. Two cancer cell lines (HT-1080 A-A1, HeLa B-B1) and normal liver BRL3A cells (C-C1) were seeded at a density of 15,000 cm^-2^, left to adhere for 6–8 h and treated with XTEN-Killin (10 μM). Cell growth and apoptosis level (annexin A5 staining) were measured. Dashed lines represent cell density and amount of apoptotic cells in the cultures at the time of plating (0 h). Statistical difference of cell density means were analyzed using one-way ANOVA with Tukey’s multiple comparison test; * p<0.05, ** p<0.01, *** p<0.001.

To obtain a more detailed picture of the effect of XTEN-Killin on cellular growth, flow cytometry after treatment with 10 μM protein was performed each 24 h for 4 days. Besides HT-1080 cells, another cancer cell line with high basal expression of MMP-2, HeLa [28] and, noncancerous BRL-3A cells, which are derived from normal rat liver, were chosen for broader examination. Growth of both cancer cell lines expressing MMP-2 and MMP-9 was stopped, and the cell number was reduced at 24 h and remained low during an observation period of 96 h. (Fig 5A and 5B). The remaining cells developed substantial apoptosis that peaked at about 60% after 48 h for HT-1080 cells (Fig 5A1) and after 72 h for HeLa cells (Fig 5B1). In contrast, normal liver cells showed growth similar to controls without substantial signs of apoptosis during the 4-day observation period (Fig 5C and 5C1).

## Discussion

The goal of the project was to demonstrate the feasibility of developing a cytostatic, activatable prodrug, including a macromolecule for long blood circulation, that can be completely produced in *E*. *coli*. For that purpose we designed a fusion protein comprising a natural cytostatic/cytotoxic element, a protease-deactivation/activation feature, a cell-penetrating peptide, and XTEN for long circulation, tumor accumulation, and additional attenuation of CPP.

Two steps were crucial in designing this fusion protein: incorporation of the XTEN polypeptide as expressible variant of PEG and deactivation of Killin, which is otherwise difficult to express in *E*. *coli*. Cho *et al*. have reported, that Killin expression (tagged with 6 histidines) in *E*. *coli* is very poor due to direct binding of the protein to the host DNA and inhibition of bacterial growth already within 30 min of induction [19]. Moreover, subsequent purification was almost impossible, since the His-tagged Killin appeared not to bind to Ni-NTA columns. Here we could demonstrate,that fusion of Killin with the XTEN polypeptide together with a CPP and an MMP-2 cleavage site enables expression and purification of the recombinant construct as a prodrug in *E*. *coli*.

Thus our hypothesis (Fig 1) that the good shielding properties of XTEN [13] together with the CPP would at least partially shut off the cytotoxicity of Killin is supported by our results. While the variant of XTEN-Killin carrying XTEN at the N-terminus was successfully expressed, there was no expression of two constructs with XTEN at the C-terminus, possibly because synthesized Killin fragments without protection by XTEN broke down during synthesis (data not shown, sequence given in Supplements). Contrary to the reported difficulty in purifying XTEN with a His-tag [19], the XTEN polypeptide synthesized here could be pre-purified by a heat step, which already removed a large part of impurities by precipitation, followed by purification of XTEN-Killin with anion exchange chromatography using the high content of 17% glutamic acid of XTEN as purification tag. Possible XTEN fragments from incomplete synthesis or partial degradation were removed by a hydrophobic interaction column (Fig 2).

Of particular importance is the activation of XTEN-Killin by MMP-2 expressing tumor or tumor stromal cells. Using protein gel electrophoresis, we confirmed the intact cleavage motive for MMP-2, which retained its function despite shielding by XTEN (Fig 2C). As cell-penetrating peptides (CPP) for uptake of the Killin fragment into tumor cells we added a polyarginine chain in conjunction with a glutamic acid chain. Activatable CPPs have been shown to be effective in enzyme-specific *in vivo* imaging of tumors [29].

Indeed, fluorescent XTEN-Killin-6S-IDCC was rapidly taken up into endosomal structures of cells with high MMP-2 expression, while uptake into cells with low MMP-2 low expression was substantially delayed (Fig 3). Unfortunately, very few cells showed good intranuclear enrichment and were apoptotic. After repeating the experiments under various conditions, we reasoned that the poor effectiveness might be attributable to the 6 sulfonic acid groups of the 6S-IDCC dye (IC Discovery GmbH). The negative charges of the dye that is coupled near the C-terminal end of the Killin and arginine chain possibly hinder binding of the activated Killin fragment to the DNA and also reduce the CPP effect of the arginines resulting in only moderate cell growth inhibition. Therefore, we coupled a fluorescein to XTEN-Killin, which is smaller and has only one negative charge. However, cells treated with XTEN-Killin-FITC construct have not shown any reduction of the growth rate either.

Ultimately, XTEN-Killin without coupled dye has demonstrated strong cell growth inhibition and massive apoptosis induction in a concentration-dependent manner 24–96 h after treatment, which is consistent with the results obtained by Cho *et al*. using a Killin peptide [19]. Taken together, the cytostatic and cytotoxic function of the activated Killin fragment appears to be highly susceptible to modifications at C-terminus, which likely affect binding to its DNA binding site.

Recently, studies analyzing neoplastic tissues of patients with prostate cancer emphasized the importance of nuclear Killin expression for reduced tumor progression and disease outcome. In addition, Killin, acting as a tumor suppressor, is down-regulated in various malignant breast carcinomas, especially in late stages [20]. Moreover, alterations in Killin genome, especially through methylation, are associated with endometrial, thyroid, and breast cancer [17,30]. Killin is a known p53 downstream target gene, inducing S-phase cell cycle arrest [19]. However, the pathway of Killin-induced apoptosis seems to be independent of p53: although overexpression of Killin leads to elevated levels of p53 and p73, cell cycle arrest occurs before, and p53-negative cells also undergo S-phase arrest and apoptosis after Killin overexpression [20].

Thus, we believe that Killin has strong potential to be used for the design of cytostatic agents. Our results with the XTEN-fused Killin fragment presented in this study, which bears a cell-penetrating peptide and enzyme-specific cleavage site, is able to arrest cancer cell growth and induce apoptosis *in vitro*, while normal liver cells and cells without MMP-2 expression are almost unaffected. Further investigations are required to evaluate XTEN-Killin *in vivo*. Aspects that should be optimized are the coupling strategy of dyes or nuclear labels for *in vivo* tracking and in histology. Furthermore, the optimal XTEN length for expression yield and cancer treatment, which is likely a longer variant, e.g., of 864 amino acids, needs to be investigated in terms of kinetics, biodistribution, and accumulation in tumors.

## Conclusions

We demonstrated the feasibility of complete expression of a fusion protein in *E*. *coli* that contains a cytostatic/cytotoxic element, a protease deactivation/activation element, a CPP and hydrophilic polypeptide for long circulation. Two crucial steps in designing this fusion protein were the deactivation of Killin and the use of the novel XTEN polypeptide, an expressible alternative to PEG. This new strategy could ultimately lead to new smart cancer drugs with reduced side effects, which could be efficiently produced in *E*. *coli* and have long blood circulation and duration of action—both advantageous for translation into the clinics.

## Supporting Information

S1 File The amino acid sequence of XTEN-Killin. Purification of XTEN-Killin (Figure A). Cleavage of XTEN-Killin with MMP-2 (Figure B). Schematic representation of XTEN-Killin DNA constructs (Figure C). Amino acid sequence of fusion protein B (,XTEN-Killin‘) (Figure D).
(PDF)
