# Supplementary material for: XTEN as Biological Alternative to PEGylation Allows Complete Expression of a Protease-Activatable Killin-Based Cytostatic
Source: PLoS One. 2016 Jun 13;11(6):e0157193. doi: 10.1371/journal.pone.0157193 (PMC4905650; doi:10.1371/journal.pone.0157193)
Supplement: S1 File — Purification of XTEN-Killin (Figure A). Cleavage of XTEN-Killin with MMP-2 (Figure B). Schematic representation of XTEN-Killin DNA constructs (Figure C). Amino acid sequence of fusion protein B (,XTEN-Killin‘) (Figure D). (PDF) [file pone.0157193.s001.pdf]

Supporting Information

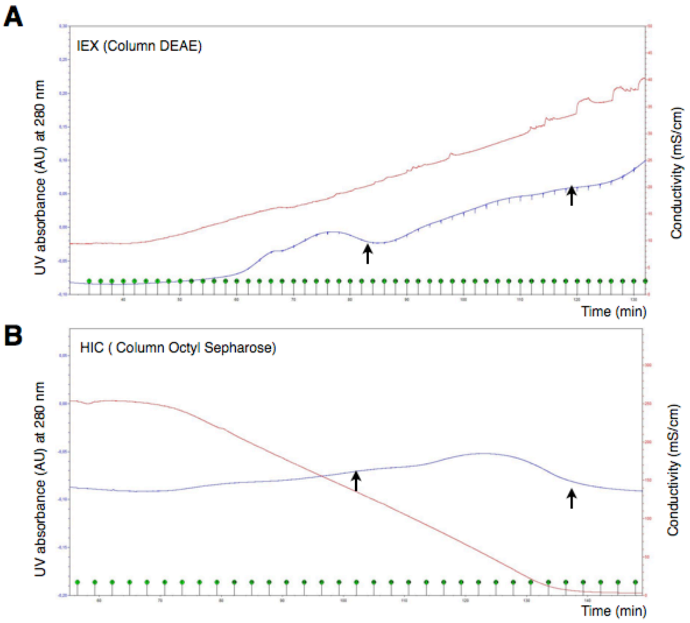

Figure A. Purification of XTEN-Killin. Chromatograms of anion exchange column (DEAE, A) and hydrophobic interaction column (Octyl Sepharose, B) demonstrate two purifications steps. Peaks between black arrows represent XTEN-Killin UV absorbance at 280 nm (blue line) during elution with sodium salt (gradient line is shown in red).

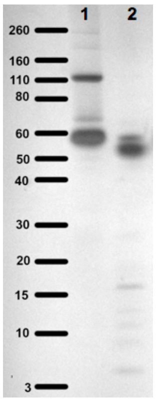

Figure B. Cleavage of XTEN-Killin with MMP-2. XTEN-Killin analyzed with SDS-PAGE (non-reducing) and silver stain before (1) and after (2) digestion with MMP-2 enzyme to prove the functionality of the cleavage site. Cleavage of monomeric (about 60 kDa) and dimeric XTEN-Killin (about 120 kDa) resulted in free XTEN polypeptide (about 55 kDa), CPP killin (approx. 5 kDa), and potential complexes formed by the arginine-rich composition of the CPP Killin are visible (approx. 17 kDa). There are some additional thin bands that could represent degradation products after digestion. Markers in A, B and D: 3 - 260 kDa.

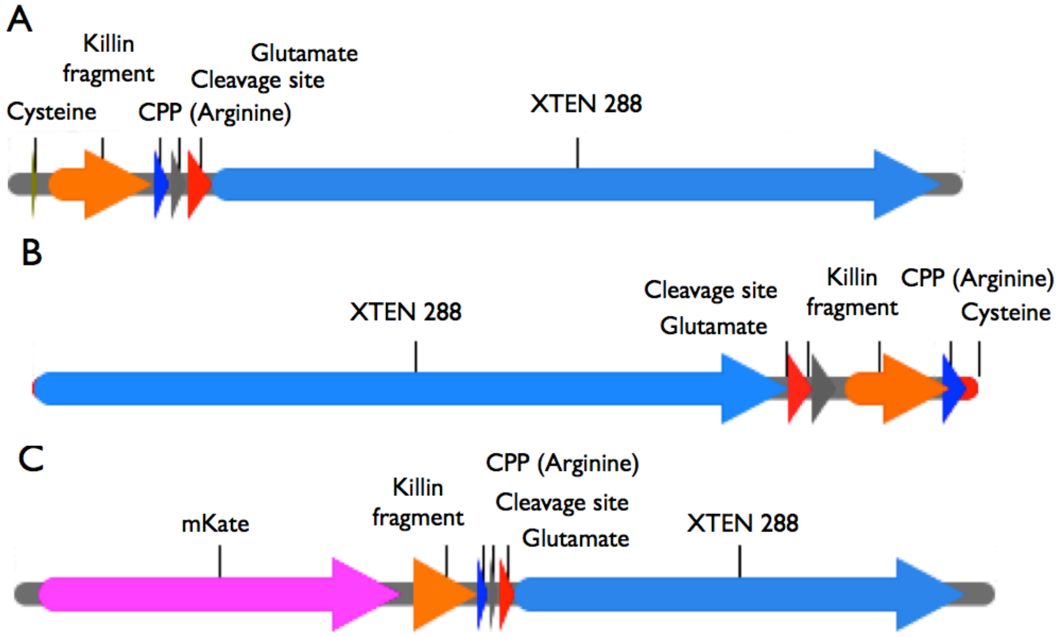

Figure C. Schematic representation of XTEN-Killin DNA constructs. Complete expression of the fusion protein in *E. coli* was accomplished only for the sequence with XTEN on the N-terminus (B).

Figure D. Amino acid sequence of fusion protein B (‚XTEN-Killin'):

MGTSESATPESGPGSEPATSGSETPGTSESATPESGPGSEPATSGSETPGTSESATPESGPGTSTEPSEGS
APGSPAGSPTSTEEGTSESATPESGPGSEPATSGSETPGTSESATPESGPGSPAGSPTSTEEGSPAGSPTST
EEGTSTEPSEGSAPGTSESATPESGPGTSESATPESGPGTSESATPESGPGSEPATSGSETPGSEPATSGSE
TPGSPAGSPTSTEEGTSTEPSEGSAPGTSTEPSEGSAPGSEPATSGSETPGTSESATPESGPGTSTEPSEG
SAPEEEEEEEEEGPLGLYLPGSARPGRTVHVWGYRVEWKVRNGRKLQPSEWAGRGDLGGFKRRRR
RRRRGGC
